# Mobile Phone-Based Measures of Activity, Step Count, and Gait Speed: Results From a Study of Older Ambulatory Adults in a Naturalistic Setting

**DOI:** 10.2196/mhealth.5090

**Published:** 2017-10-03

**Authors:** Cassia Rye Hanton, Yong-Jun Kwon, Thawda Aung, Jackie Whittington, Robin R High, Evan H Goulding, A Katrin Schenk, Stephen J Bonasera

**Affiliations:** ^1^ Department of Internal Medicine, Division of Geriatrics University of Nebraska Medical Center Omaha, NE United States; ^2^ Department of Physics Randolph College Lynchburg, VA United States; ^3^ Department of Biostatistics College of Public Health University of Nebraska Medical Center Omaha, NE United States; ^4^ Department of Psychiatry and Behavioral Sciences Northwestern University Chicago, IL United States

**Keywords:** mobile phone, functional status, mobility, gait speed, mobility measures, LLFDI, SAFFE, PROMIS short, PROMIS Global, step count, behavioral classification, frailty phenotype, normal aging

## Abstract

**Background:**

Cellular mobile telephone technology shows much promise for delivering and evaluating healthcare interventions in cost-effective manners with minimal barriers to access. There is little data demonstrating that these devices can accurately measure clinically important aspects of individual functional status in naturalistic environments outside of the laboratory.

**Objective:**

The objective of this study was to demonstrate that data derived from ubiquitous mobile phone technology, using algorithms developed and previously validated by our lab in a controlled setting, can be employed to continuously and noninvasively measure aspects of participant (subject) health status including step counts, gait speed, and activity level, in a naturalistic community setting. A second objective was to compare our mobile phone-based data against current standard survey-based gait instruments and clinical physical performance measures in order to determine whether they measured similar or independent constructs.

**Methods:**

A total of 43 ambulatory, independently dwelling older adults were recruited from Nebraska Medicine, including 25 (58%, 25/43) healthy control individuals from our Engage Wellness Center and 18 (42%, 18/43) functionally impaired, cognitively intact individuals (who met at least 3 of 5 criteria for frailty) from our ambulatory Geriatrics Clinic. The following previously-validated surveys were obtained on study day 1: (1) Late Life Function and Disability Instrument (LLFDI); (2) Survey of Activities and Fear of Falling in the Elderly (SAFFE); (3) Patient Reported Outcomes Measurement Information System (PROMIS), short form version 1.0 Physical Function 10a (PROMIS-PF); and (4) PROMIS Global Health, short form version 1.1 (PROMIS-GH). In addition, clinical physical performance measurements of frailty (10 foot Get up and Go, 4 Meter walk, and Figure-of-8 Walk [F8W]) were also obtained. These metrics were compared to our mobile phone-based metrics collected from the participants in the community over a 24-hour period occurring within 1 week of the initial assessment.

**Results:**

We identified statistically significant differences between functionally intact and frail participants in mobile phone-derived measures of percent activity (*P*=.002, t test), active versus inactive status (*P*=.02, t test), average step counts (*P*<.001, repeated measures analysis of variance [ANOVA]) and gait speed (*P*<.001, t test). In functionally intact individuals, the above mobile phone metrics assessed aspects of functional status independent (Bland-Altman and correlation analysis) of both survey- and/or performance battery-based functional measures. In contrast, in frail individuals, the above mobile phone metrics correlated with submeasures of both SAFFE and PROMIS-GH.

**Conclusions:**

Continuous mobile phone-based measures of participant community activity and mobility strongly differentiate between persons with intact functional status and persons with a frailty phenotype. These measures assess dimensions of functional status independent of those measured using current validated questionnaires and physical performance assessments to identify functional compromise. Mobile phone-based gait measures may provide a more readily accessible and less-time consuming measure of gait, while further providing clinicians with longitudinal gait measures that are currently difficult to obtain.

## Introduction

Across a variety of medical disciplines, longer-term measures of gait performance have the potential to benefit both patients and practitioners. Gait speed remains an underutilized clinical measure, despite convincing data suggesting that decreases in gait speed are associated with greater mortality [1], diminished cognition [2], greater functional disability [3], poorer quality of life, and increased healthcare spending [3,4]. There is also evidence suggesting that improved gait speed may be a sensitive biomarker for improved overall functional status [5]. Resistance to including gait speed in current clinical practice is multifactorial, with time and space constraints and provider unfamiliarity being major factors [6]. Longitudinal clinical measures of gait speed are also challenging to obtain, since collecting these measures may be more subject to various biases than more easily obtained metrics such as pulse oximetry or body weight [7].

In the past, gait speed studies have typically relied on measurements taken in the clinic. The standard method for determining gait speed involves timing an individual while walking a short, predetermined distance (eg, 4 to 6 meters). This approach is less than ideal because physical activity, including gait, is influenced by performance biases (eg, participants who know they are being observed try to improve their usual performance), as well as ultradian, circadian, and seasonal changes that cannot be evaluated during a single clinic visit [8]. Furthermore, in older persons, gait speed declines slowly over long periods of time, necessitating repeat observations [9,10].

The rise of widely used electronic devices, such as mobile phones with app capabilities (smartphones), offers great potential for remote monitoring of patient gait speed and other clinically relevant health parameters. We have shown the feasibility of using mobile phone technology to measure an individual’s activity and lifespace (eg, the geographic expanse of an individual’s day-to-day travels) over prolonged periods of time in a non-invasive, near-continuous, robust, inexpensive, and user-friendly manner [11]. In order to extrapolate health parameters [12] from our participant-derived mobile phone data, we designed algorithms to measure clinically relevant aspects of activity, including gait bout duration, gait speed, and step counts [13]. Additional studies showed that for a broad group of individuals (ranging in age from 21 to 84 years), the activity metrics we measured by this approach strongly correlated with gait speed under controlled laboratory conditions [14].

Here, we show for the first time that mobile phones can provide both continuous and aggregate measures of clinically relevant gait and mobility parameters, including gait speed, step count, and overall activity status, in a community dwelling population going about their day-to-day lives. We gave participants a mobile phone, instructed them in its use, and recorded their activities over the next 24 hours. Validated algorithms were used to classify this data into clinically relevant gait parameters. We studied both healthy (eg, functionally intact) and frail (eg, functionally impaired) community dwelling older individuals. Frailty is a clinical syndrome characterized by poor activity tolerance, weakness, and weight loss unexplained by known diseases of the muscle and brain; frailty is a particularly significant problem leading to greater morbidity and mortality and poorer quality of life [15,16]. Our results suggested that mobile phone-derived measures of these parameters differentiated between older adults without functional limitations and older adults with a frailty phenotype. These mobile phone-derived measures also assessed aspects of functional status distinct from those quantified by either a number of validated questionnaire tools or standard clinical physical performance measures.

## Methods

### Participant Enrollment Procedures

**Figure 1:**
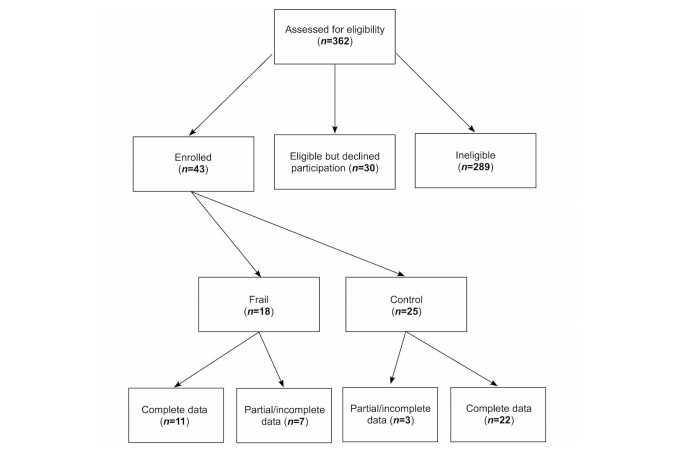
Enrollment flow diagram.

Participants for this study were recruited from the University of Nebraska Medical Center (UNMC) Geriatrics Clinic and the Engage Wellness Center, both part of UNMC’s Home Instead Center for Successful Aging (HICSA). We assembled 2 ambulatory cohorts: one of healthy older individuals with no functional impairment (n=25), and one of frail [17] older individuals (n=18). For our functionally intact group, inclusion criteria were (1) age 55 or older; (2) community dwelling; (3) no serious uncontrolled medical or psychiatric comorbidities; and (4) a minimum score of 23 out of 30 on the Mini-Mental State Examination (MMSE) [18] or Montreal Cognitive Assessment (MoCA) [19]. For our frail group, inclusion criteria also required having 3 of the 5 following clinical conditions present at enrollment: (1) less than 10% unintentional weight loss or body mass index (BMI) less than 18.5 kg/m^2^; (2) slow (less than 0.8 m/s) walking speed [20]; (3) weak grip strength (measured by a hand dynamometer, JAMAR, Bolingbrook, IL); (4) reports of exhaustion; and (5) low activity. Of note, the cognitive criteria required that we screened a large number of potential participants for our frailty group. The UNMC Institutional Review Board approved this study. Written informed consent was obtained from all participants. The enrollment flow diagram is shown in Figure 1 and the baseline cohort characteristics are shown in Table 1.

### Self-Reported Functional Status

We used previously validated survey instruments to determine participant self-perceived functional status. These instruments included the (1) functional component of the Late Life Function and Disability Instrument (LLFDI), a comprehensive assessment of function and disability for use in community-dwelling older adults that evaluates self-reported difficulty performing 32 physical activities (eg, use of a stepstool or running to catch a bus), where higher scores indicate higher functional status) [21]; (2) the Survey of Activities and Fear of Falling in the Elderly (SAFFE), a questionnaire evaluating fears associated with performing 11 activities of everyday life (eg, if the participant is limited going to the store or going out when it is slippery) necessary for independent living [22]; (3) Patient Reported Outcomes Measurement Information System (PROMIS) Global Health (GH), short form version 1.1 [23], subdivided into assessments of physical health (PROMIS-PH) and mental health (PROMIS-MH); and (4) PROMIS short form version 1.0 Physical Function 10a (PROMIS-PF). These PROMIS outcome measures were designed to assess patient experience of health outcomes such as pain, fatigue, physical function, depression, anxiety, and social function [24,25]. PROMIS instruments are based on strong psychometrics and consequently have fewer problems with floor and ceiling effect than other survey instruments.

Participants were comfortably seated in a quiet room to complete the above questionnaires, which were administered by a tablet computer (iPad, Apple Inc.). All questionnaire results were stored using the Research Electronic Data Capture (REDCap) database [26]. Participants were given as much time as they needed to complete the surveys. Staff provided no assistance during this process and participants had to complete all the questions to remain eligible for the study.

**Table 1:** Baseline cohort characteristics (N=40).

Characteristic		Functionally-intact, n (%)	Frail, n (%)	*P*
Overall		22 (61%)	18 (39%)	
**Gender**				.606
	Female	17 (77%)	12 (67%)	
	Male	5 (23%)	6 (33%)	
				
**Age, years**				<.001
	50-60	1 (5%)	N/A	
	61-70	5 (23%)	1 (6%)	
	71-80	14 (63%)	5 (28%)	
	81-90	2 (9%)	7 (39%)	
	91-100	N/A	5 (28%)	
				
**Ethnicity**				.37
	Non-Hispanic white	20 (91%)	17 (94%)	
	Other	2 (9%)	1 (6%)	
				
**Current residence**				
	Home (rented or owned)	19 (86%)	13 (72%)	.26
	Apartment	2 (9%)	4 (22%)	.25
	Assisted living facility	N/A	1 (6%)	.33
	Other	1 (5%)	N/A	.33
				
**Living with**					
	Alone	14 (64%)	8 (44%)	.17
	Spouse or significant other	6 (27%)	4 (22%)	.80
	Spouse with children, caregiver	N/A	4 (22%)	.04
	Other	2 (9%)	2 (11%)	.80
				
**Education**				<.001
	Grades 9-11	1 (5%)	2 (11%)	
	Grade 12 or GED^a^	2 (9%)	5 (28%)	
	College 1-3 years	8 (36%)	5 (28%)	
	College 4 years	5 (23%)	4 (22%)	
	Graduate school	6 (27%)	2 (11%)	
				
**BMI^b^**				.934
	<20	1 (5%)	1 (6%)	
	20-25	6 (27%)	4 (22%)	
	26-30	9 (41%)	5 (28%)	
	31-35	5 (23%)	6 (33%)	
	35+	1 (5%)	2 (11%)	

^a^GED: general education development.

^b^BMI: body mass index.

### Clinical Gait Measures

All participants underwent a 4 meter walking test [27] consisting of a 1 meter untimed startup followed by a 4 meter timed evaluation. Participants were given the instruction to “walk at your usual speed” and were permitted to use an assistive device such as a walker or cane at their discretion. Participants then performed a 10 foot “Get Up and Go” test [28]. They began the test seated with their back against the backrest of an armless chair. They were instructed to stand up and “walk at your usual speed” to a mark 10 feet directly in front of the chair, turn around, return to the chair, and sit down again. Timing stopped when their back once again touched the backrest of the chair. Finally, we video recorded participant performance during a Figure-of-8 Walk (F8W) [29]. The camera focused on the participant’s lower legs and feet during the test. No identifying features were photographed. Participants were instructed to walk in a figure-of-8 at their self-selected pace around 2 cones placed 5 feet apart. Total completion times, the number of steps to complete the F8W, and gait smoothness were recorded. Two trials of all physical assessment tests were performed. Participants tolerated all of these clinical assays with ease.

### Gait Data Acquisition

Nokia N79 mobile phones (White Plains, NY) with an intrinsic three-dimensional (3D) accelerometer were used to measure mobility and locomotion for extended periods of time in community dwelling individuals of both cohorts. Acceleration values were sampled and written to memory using custom Python software (Python for S60 v1.9.7) [30], running on a Symbian S60 V3FP2 OS (San Francisco, CA). The mobile phone was placed in either the participant’s right or left pocket, over the hip, and the location recorded. A previous study of ours showed that location did not impact data collection [14].

### Protocol

Participants were fitted with a mobile phone; the proper use and correct placement of this device was demonstrated. Participants were instructed to wear the mobile phone for the next 24 hours, except when bathing, swimming, or sleeping.

Participants were then briefly videotaped walking on a treadmill (SCIFIT, Tulsa, OK) for 5 minutes at 2 mi/hr or a speed more comfortable for the individual participant. Participants unable to walk on the treadmill due to limited mobility, the need for assistive devices, or other factors, were not asked to complete this portion of the study. Gait speed calculations depended upon stride length, which we derived from treadmill locomotion videos (1.38 m functionally intact; 0.83 m frail).

### Data Quality Control and Classification

Survey data was scored per instrument instructions. For PROMIS measures, *t* scores were determined from raw scores by appropriate conversion tables. Raw acceleration data was low-pass filtered and baseline acceleration normalized to 1 g over the entire duration of data collection [13]. Our classification algorithm first identified epochs of “forgotten phone” versus epochs of participant carrying the phone. For epochs of participant carrying the phone, we then classified behavior into active or inactive states, using a windowed (68 s long) Fourier analysis approach [13,31]. Active states were further differentiated into states with minimal locomotion, states with ongoing locomotion, and states where the participants were climbing stairs. Ongoing locomotion was then quantified for step count and gait speed. Gait speed calculations depended on treadmill video-derived values of stride length.

### Statistical Analysis

Step count, gait speed, and activity count were primary outcomes with cohort (functionally intact versus impaired) and time as factors. All comparisons not involving time were performed by 2-tailed *t* tests assuming unequal variances. Multiple comparisons were adjusted using the Bonferroni technique. For comparisons over time, we performed repeated measures analysis of variance (ANOVA) including all interaction terms; interactions not found to be significant were dropped from later models. All post hoc testing was performed using Tukey test. To measure pairwise agreements between mobile phones, surveys, and physical performance battery-based functional measures, we evaluated Bland-Altman plots using MATLAB (blandaltman.m). For gait speed measures, we performed a bootstrap analysis using MATLAB (datasample) to determine statistical significance between the functionally intact and frail cohorts. Functional questionnaire data and clinical physical performance measures were analyzed by 1-way ANOVA. Spearman correlations were determined to assess agreements between mobile phone-based measures and the survey- and/or performance-based metrics. Finally, cohort demographic factors were compared using independent samples *t* test assuming equal variances (2-tailed). All analyses were performed using SPSS (IBM SPSS Statistics 22.0, Armonk, New York, USA) or MATLAB (R2011b, MathWorks, Natick, MA).

## Results

A total of 362 participants were assessed for study eligibility (Figure 1) and, from those, 73 (20.2%, 73/362) were identified as potential study participants. Of those, 30 (41%, 30/73) individuals declined participation and 43 (59%, 43/73) were consented into the study. Of the 43 participants enrolled in the study, 25 (58%, 25/43) were enrolled in the control (functionally intact) arm and 18 (42%, 18/43) were enrolled in the frail (functionally impaired) arm. Unfortunately, mobile phone sensor data was not collected from 10 participants (23%, 10/43) in the functionally intact arm and 7 (16%, 7/43) from the frail arm due to technical problems with data transmission and storage. By far, the greatest challenge we encountered during participant recruitment was identifying frail individuals with preserved cognition.

During our 24-hour study period, all 22 functionally-intact participants recorded at least 14 hours of data, with a mean of 17.3 hours (range 14 to 20 hours) for a total of 380 hours suitable for analysis. We determined that 11 (61%, 11/18) participants from the frail arm recorded at least 9 hours of data, with a mean of 19.9 hours (range 9 to 24 hours) for a total of 210 hours, of which 209 were suitable for analysis (1 hour prematurely truncated). There was no significant difference in the number of hours recorded for the functionally intact versus frail cohort (*P*=.17) when normalized over the 24-hour day. We obtained similar amounts of data from both functionally intact and frail older individuals. Baseline demographics between the 2 groups revealed that our functionally intact participants were younger and had higher educational achievement compared to our frail participants; cohorts were otherwise comparable regarding gender, ethnicity, housing, and BMI (Table 1).

### Gait Assessment Survey Instruments and Clinical Performance Measures

We chose our questionnaires and performance assessments based on prior validation, current clinical and/or research use, and face validity. In total, 40 (93%, 40/43) participants successfully completed the questionnaires and physical performance batteries. As expected, our analysis demonstrated that LLFDI, SAFFE, PROMIS-PH, and PROMIS-PF all differentiated individuals from functionally intact and frail groups (Table 2). Our 3 clinical physical performance measures (10-foot timed Get up and Go, 4 Meter Walk, and F8W) also performed as expected (Table 2), with all measures demonstrating robust differences between our functionally intact and frail cohorts. The LLFDI scores range between 0 (full limitations for performing tasks) to 100 (no limitations for performing tasks) [32]. SAFFE activity level scores ranged between 0 (lowest function) and 11 (highest function), SAFFE fear of falling scores range between 0 (no fear of falling) and 3 (high fear of falling), and SAFFE activity restriction scores range between 0 (no activity restrictions) and 11 (marked activity restrictions) [33]. PROMIS *t* scores set average performance for a US-based population at 50 (SD 10 points), with better function indicated by higher scores. Scoring was performed per PROMIS-GH and PROMIS-PF [34]. EuroQol scores were derived from PROMIS-GH, and range between 0 (very poor health-related quality of life) and 1 (very high health-related quality of life).

**Table 2:** Statistical significance of standard questionnaire and physical performance battery in discriminating functionally impaired from functionally intact participants.

Survey instrument		Intact^a^	Impaired^a^	*P*^b^
**Questionnaire**				
	LLFDI^c^ Overall Function	64.04	46.91	<.001
	LLFDI basic lower extremity function	76.02	54.23	<.001
	LLFDI advance lower extremity function	56.15	22.41	<.001
	LLFDI upper extremity function	79.57	68.95	.257
	SAFFE^d^ activity level	9.32	6.38	<.001
	SAFFE fear of falling	0.24	0.39	.052
	SAFFE activity restriction	2.45	6.59	<.001
	PROMIS^e^-PF^f^	48.94	34.43	<.001
	PROMIS-PH^g^	51.99	40.52	<.001
	PROMIS-MH^h^	63.65	55.79	.042
	EuroQol	0.764	0.64	<.001
**Performance battery^i^**					
	Timed Get Up and Go (10 ft)	10.63	21.79	.003
	4 Meter Walk	4.30	10.66	.004
	Figure-of-8 Walk	9.19	19.28	.008

^a^Mean performance.

^b^*P* values are 2-sided *t* test, unequal variance, with Bonferroni correction.

^c^LLFDI: Late Life Function and Disability Instrument.

^d^SAFFE: Survey of Activities and Fear of Falling in the Elderly.

^e^PROMIS: Patient Reported Outcomes Measurement Information System.

^f^PROMIS-PF: PROMIS Physical Function.

^g^PROMIS-PH: PROMIS Global Physical Health.

^h^PROMIS-MH: PROMIS Global Mental Health.

^i^Values for all performance battery measures are reported in seconds.

**Figure 2:**
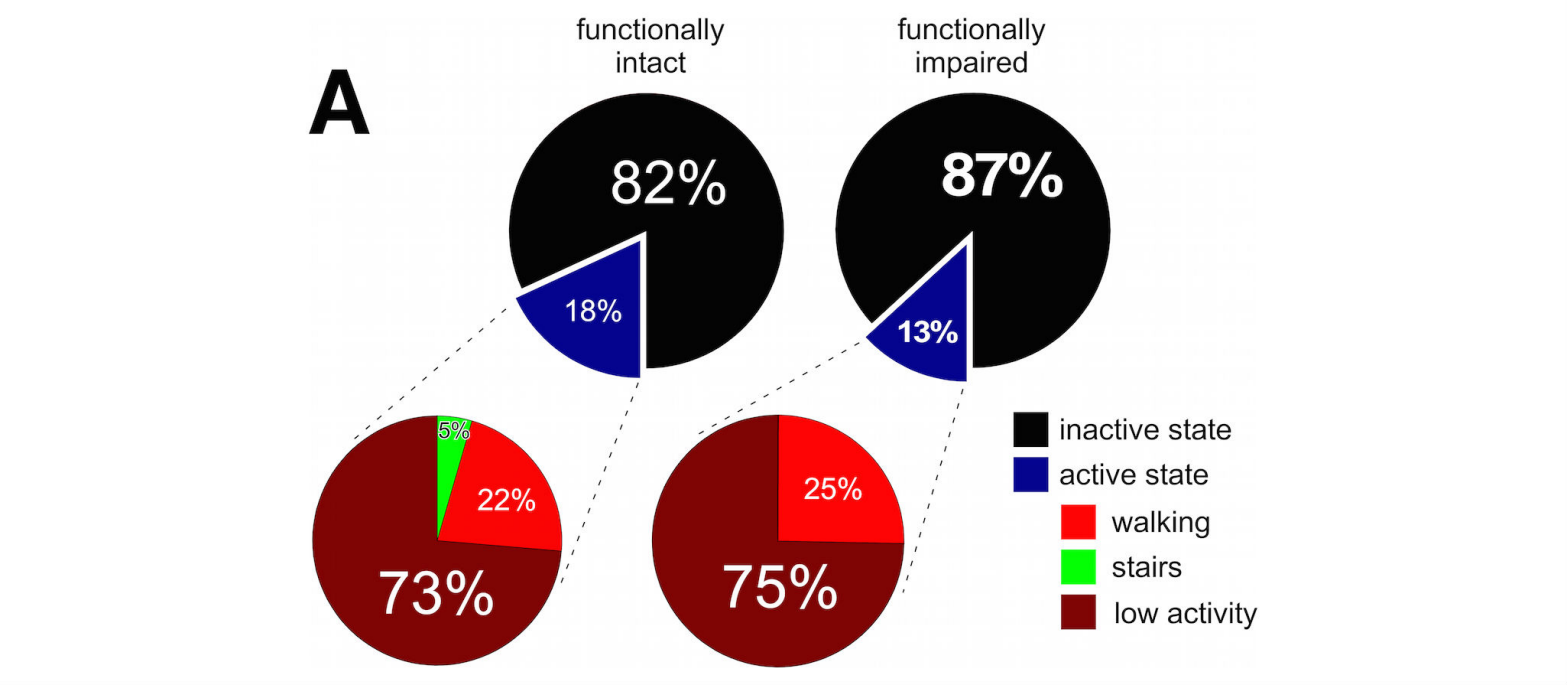
Mobile phone-derived activity metrics discriminate between frail and functionally-intact individuals. 24-hour time budget for functionallyintact (left) and functionally impaired (right) participants. Time spent in active state (blue slices) is further broken down into periods of low (brown)and high (red, green) physical activity. Percentages (bold) statistically differ between cohorts.

**Figure 3:**
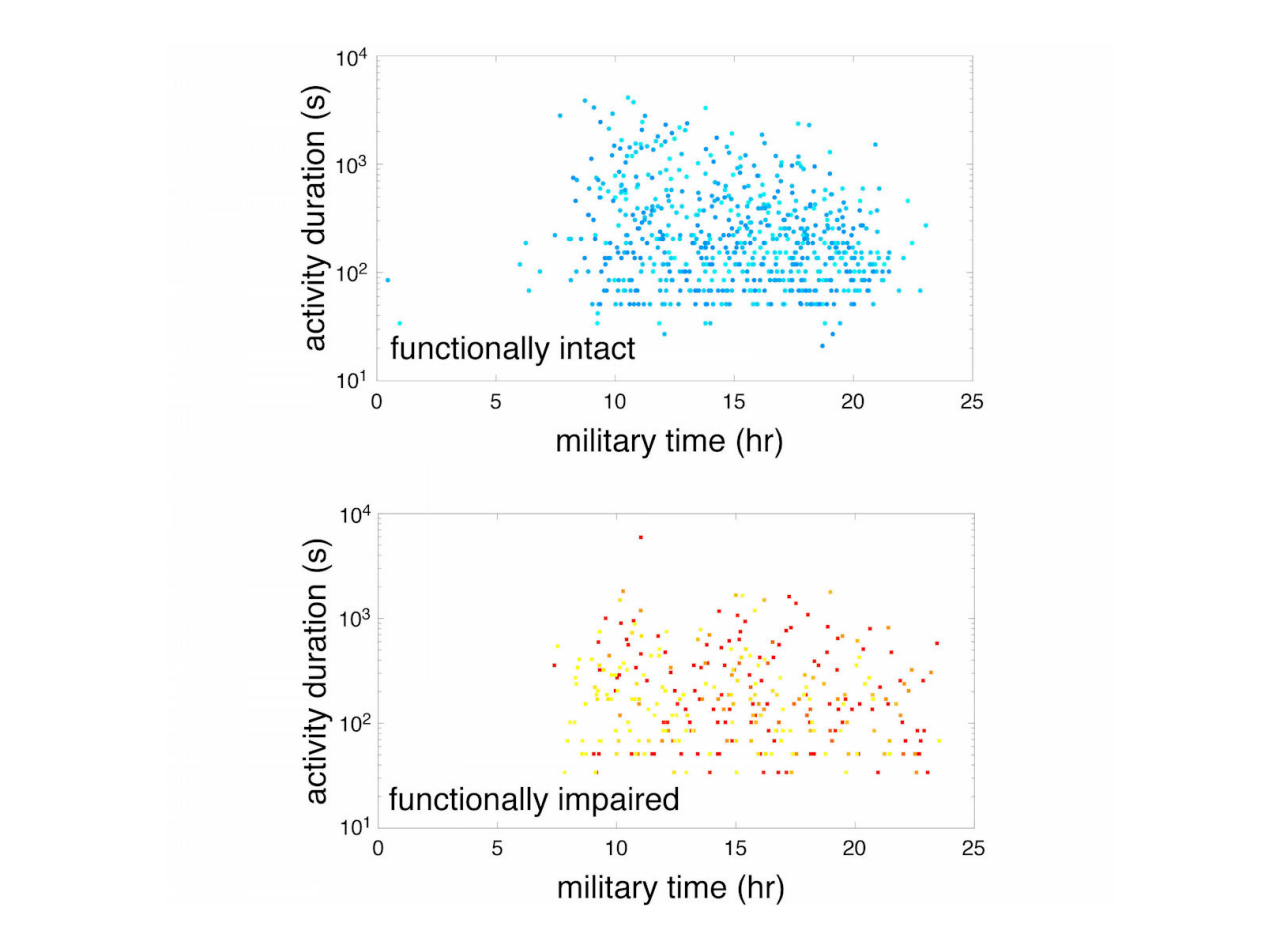
Semilog (y axis) of activity duration versus start time of that particular activity bout (x axis) in functionally intact individuals (upper) and functionally impaired individuals (lower). Each point represents a specific activity bout and each color corresponds to a specific participant ("cool" or "warm" colormap).

### Mobile Phone-Based Functional Measures

**Figure 4:**
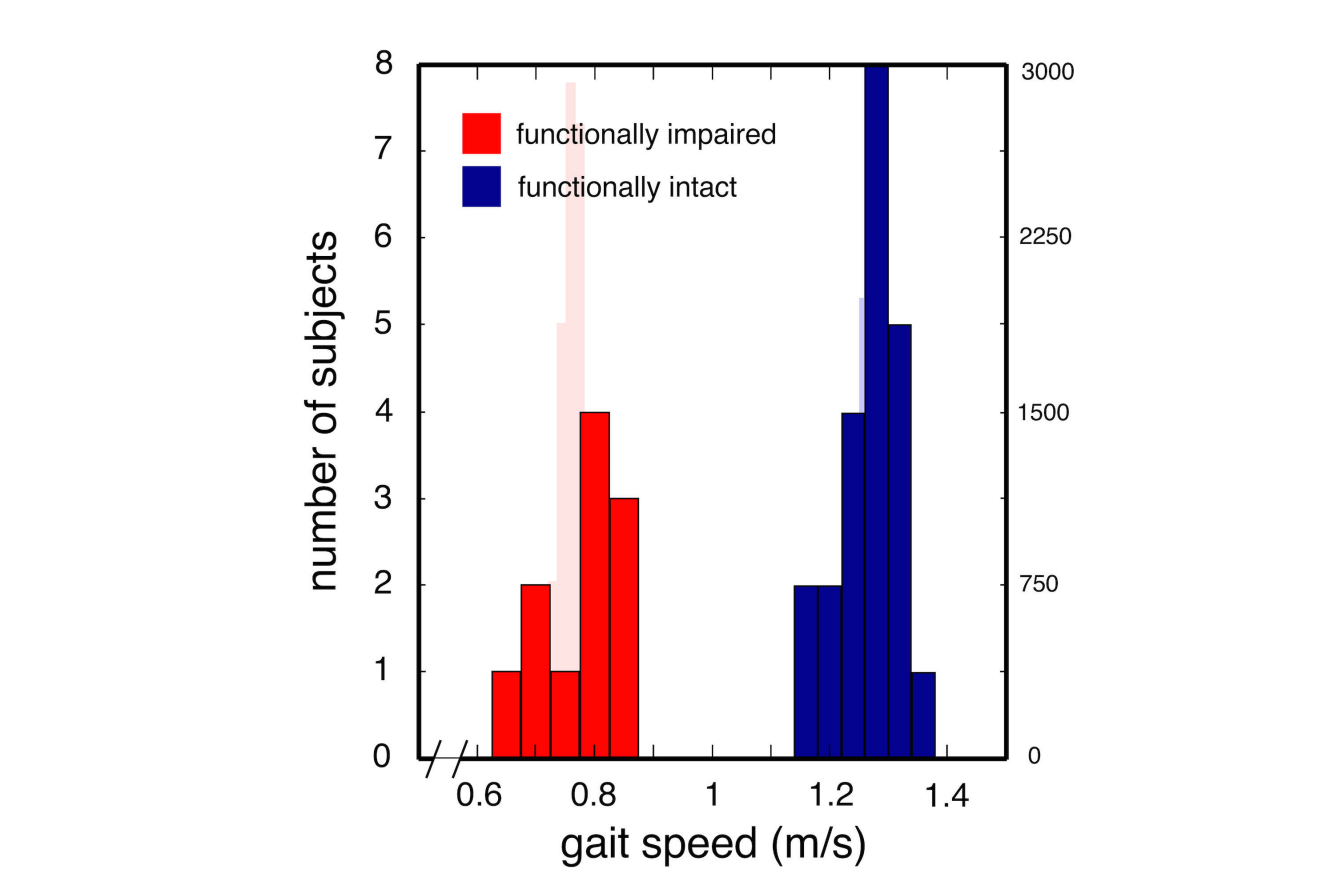
Mean daily gait speed histogram depicting significant differences between functionally intact (blue) and functionally impaired (red) participants. Bootstrap estimates of mean gait speed are provided behind data histograms (light red for functionally impaired; estimate for functionally intact group is completely behind data histogram).

After determining that our questionnaire-based measures and physical performance battery successfully differentiated functionally intact from functionally impaired individuals, we examined whether our mobile-phone-based measures of physical activity did so as well. Active states were defined as periods where the participant was walking, climbing stairs, or otherwise active (high physical activity classification) [13]. Inactive states were defined as when the participant was resting or driving (low physical activity) [13]. We noted significant differences in participant 24-hour and active state time budgets (Figure 2). Overall, the functionally intact group were active approximately 18% of the day with a mean of 18.13% (SD 5.54%); while the frail group displayed significantly less activity with a mean of 13.19% (SD 5.20%; *P*=.02 for intact versus frail groups, 2-sided *t* test). There were no phenotypic differences in active state onset rate between functionally intact and impaired individuals with 2.63 onsets/hr (SD 0.162) and 2.48 onsets/hr (SD 0.219), respectively (*P*=.60, 2-sided Student *t* test). Functionally intact individuals had longer active state durations of 373.85 s (SD 20.66) compared to frail individuals with active state durations of 300.19 s (SD 25.79; *P*=.04; 2-sided Student *t* test) (Figure 3). Similarly, average gait speed (measured over a 24-hour window) differed significantly between frail and functionally intact groups with mean gait speeds of 0.76 m/s (SD 0.08) and 1.22 m/s (SD 0.14), respectively (F_1,30_=21.1, *P*<.001) (Figure 4).

**Figure 5:**
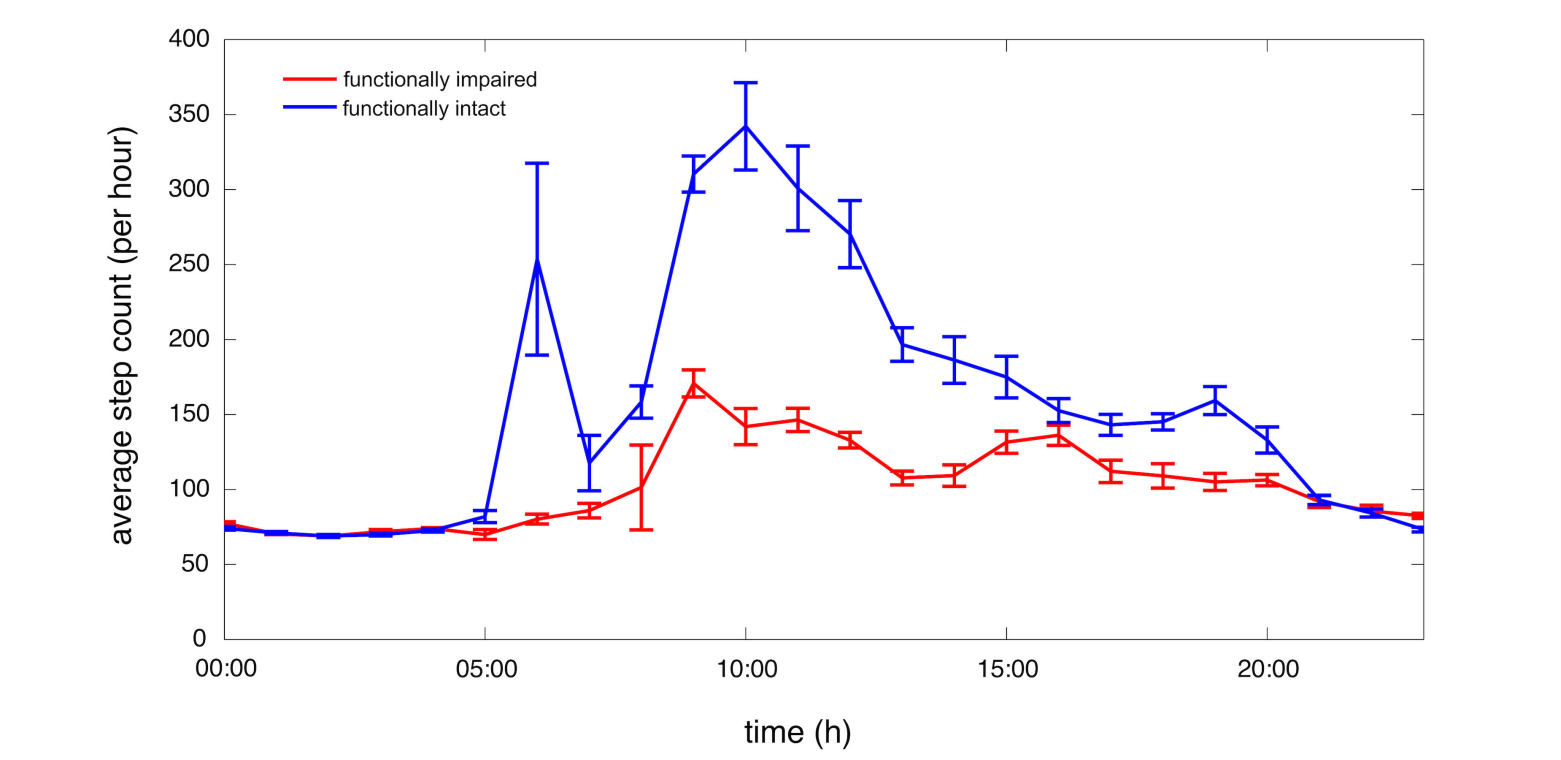
Step count versus circadian time for functionally intact (blue) and frail (red) individuals. Bars are plus or minus one standard error of the mean. Time values given in military time.

The average number of step counts throughout a circadian day also differed between frail and functionally intact groups (Figure 5). Repeated measures 2-way ANOVA, with log step count as the dependent variable and functional status and time as independent variables, found functional status, time, and the status by time interaction to be significant (F_1,30_=12.1, *P*=.002 for functional status; F_23,521_=9.0, *P*<.001 for time; F_23,521_=1.6, *P*=.045 for functional status by time interaction). Overall, all mobile phone collected outcomes, including step count, gait speed, activity classification, and percent activity were statistically significant in our study, indicating substantial differences between functionally intact and frail participants.

### Aspects of Gait Assessed by Survey and/or Performance Battery and Mobile Phone Functional Measures Assess Different Aspects of Gait

We further decided to determine (1) if our mobile phone-based measures identified similar elements of frailty as survey and physical performance assessments; and (2) if performance data obtained in this study (whether from mobile phone, survey, or performance battery) demonstrated internal consistency. First, we evaluated mean-difference (Bland-Altman) plots in a pairwise manner comparing mobile phone-, survey-, and performance battery-based functional measures. Bland-Altman plots provide a graphical approach to determine if results from two different measurement methods assessed a similar construct; if this were the case, the plotted residuals would form a relatively uniform-width band parallel to the x axis. Our analysis suggested that only LLFDI overall/LLFDI basic and PROMIS PF/PROMIS PH measured similar outcomes in both functionally intact and frail participants (Multimedia Appendix 1).

**Figure 6:**
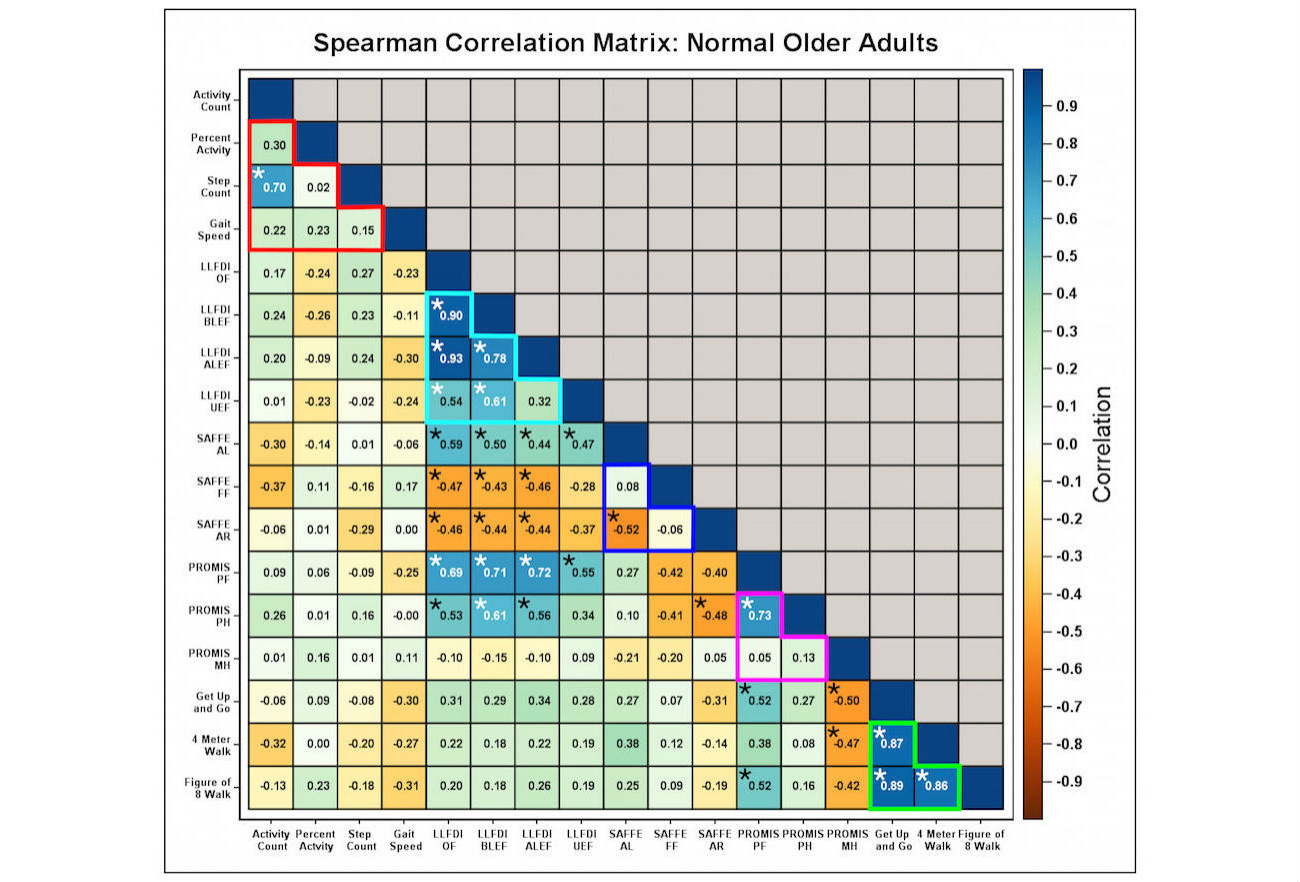
Multiple significant correlations across different functional assessment metrics are present in functionally intact older adults. Confusion matrix depicting correlation structure (metrics across matrix bottom row) of mobile phone-based activity measures (activity count, percent activity, step count, gait speed), questionnaire-based measures of functional status (LLFDI, SAFFE, PROMIS), and performance battery based measures of functional status (Get Up and Go, 4 meter walk, F8W) for functionally intact participants. For each entry, correlation strength is depicted as the color within the box; interpretation color bar provided on the right. Numeric values within each box are individual correlations. Asterisks depict interactions with P values less than .01. Interactions grouped within the red lines depict correlations within mobile phone-derived activity measures; interactions grouped within cyan lines depict correlations within LLFDI measures; interactions grouped within blue lines depict correlations within SAFFE measures; interactions within grouped violet lines depict correlations within PROMIS measures; interactions grouped within green lines depict correlations within performance battery measures.

**Figure 7:**
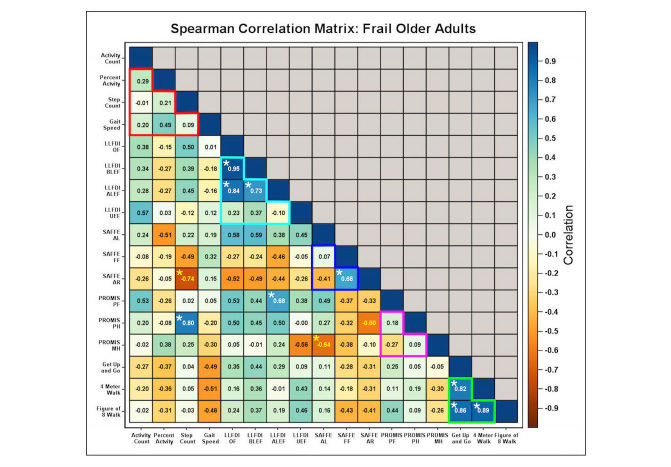
Fewer significant correlations across different functional assessment metrics are present in functionally impaired older adults. Confusion matrix depicting correlation structure of mobile phone-based activity measures, questionnaire-based measures of functional status, and performance battery based measures of functional status for frail participants. Layout similar to Figure 6.

We then calculated Spearman correlations between survey and/or performance instruments and the mobile phone-based functional measurements for functionally intact (Figure 6) and frail (Figure 7) participants. In functionally intact participants, we noted significant within-test correlations for our mobile phone-based monitoring metrics (step and activity count; 1 of 6 potential correlations), all LLFDI metrics (except for those measuring upper extremity function [UEF]; 5 of 6 potential correlations), SAFFE metrics (activity restriction and limitation; 1 of 3 potential correlations), PROMIS metrics (PROMIS-PH and PROMIS-PF; 1 of 3 potential correlations), and all performance battery results (3 of 3 potential correlations). LLFDI metrics (except UEF) also strongly correlated with results from both SAFFE and PROMIS (except PROMIS-MH; 17 of 25 potential correlations). By contrast, both within-instrument and across-instrument correlations were weaker in frail adults with functional impairment. Only performance battery and subsets of LLFDI scores remained significantly correlated with one another (3 of 3 potential correlations for functional battery metrics; 3 of 6 potential correlations for LLFDI metrics). Much of the correlation between LLFDI and SAFFE/PROMIS metrics was no longer observed. Step and activity counts no longer correlated with one another in functionally impaired individuals; however, step count now demonstrated significant correlations with both SAFFE activity restriction and PROMIS-PH.

## Discussion

### Principal Findings

To our knowledge, we present the first demonstration that mobile phones measure clinically relevant functional metrics, including overall activity, gait speed, and step count. These measures were taken over one day in naturalistic conditions and real-life settings, and thus provided insights regarding individual function outside of the clinic. We provided further validation of the LLFDI, SAFFE, PROMIS-GH, PROMIS-PF, timed 4-meter walking test, timed Get Up and Go, and F8W assays demonstrating that cognitively intact individuals with functional loss had worse performance on all of these assays compared to functionally intact individuals. In functionally intact individuals, mobile phone-based metrics and survey and/or performance battery results did not strongly correlate with one another, suggesting that these different tools measure distinct aspects of physical function. However, in cognitively intact individuals with functional loss, mobile phone-based functional metrics strongly correlated with components of both SAFFE and PROMIS. Thus, in functionally impaired individuals, mobile phone-based metrics of impaired physical function reflected parallel losses of both perceived and enabled physical function.

### Measuring Individual Functional Status Using Mobile Phones

Our study advances the goal of an easy-to-use, robust, accurate, second nature system that measures clinically relevant activity metrics (onsets, durations, step counts, and gait speeds) in different ambulatory populations. This goal is attainable with appropriate hardware and software. For example, over 50 years ago, Stunkard [35] showed the feasibility of using pedometers to estimate individual walking distance over long observations. Technical refinements (improved accelerometer technology, device durability, device data logging) have since increased data accuracy and temporal precision [36-38]. However, dedicated devices validated in many small trials to measure individual activity status have not “caught on” with the population at large, potentially because these devices did not successfully address human usability factors [39]. By contrast, mobile phones have become a nearly ubiquitous technology [40-42]. This is particularly true among younger and middle-aged adults, whose quality of life stands to significantly benefit from advances in mobile phone-based healthcare delivery and follow-up. However, we do note that while we had excellent adherence to our data collection in the functionally intact older adult group, we had less success with data collection in the frail older adult group. This decrease in adherence suggests that older adults with functional limitations may have more difficulties using this technology successfully. Devices to serve this population may require further engineering to optimize user interface features.

### Metric Validation

This study provided additional opportunity to further validate a number of questionnaire and performance instruments designed to measure functional status. The LLFDI [21] assesses two distinct outcomes: function (ability to do discrete actions or activities), and disability (performance of socially defined life tasks). Prior studies have validated LLFDI for identifying functional deficits in independent older adults [43], institutionalized older adults [44], older adults with knee osteoarthritis [45], older adults with chronic renal disease [46] and incontinence [47], and persons undergoing cardiac physical therapy [48]. LLFDI has comparable psychometric properties to performance-based measures of upper and lower extremity function [49]. Our results suggested that LLFDI can discriminate functional status between a cohort of functionally intact older adults and persons meeting frailty criteria without cognitive impairment. We also demonstrated that in functionally intact, but not frail, individuals, LLDFI is highly correlated across its functional submeasures (LLFDI basic lower extremity function [BLEF], LLFDI advanced lower extremity function [ALEF], etc), and is significantly correlated to both SAFFE and PROMIS (except PROMIS-MH) scores. For all participants, LLFDI was not significantly correlated to either mobile phone gait speed or physical performance battery measures.

SAFFE evaluates how fear of falling influences participant activity participation or restriction. It has been validated in community dwelling older adults [22,50], older adults with mobility limitations [51], and extensively utilized in studies of persons with Parkinson’s disease [52-54] as well as individuals receiving post-fall physical therapy who have a fear of falling [22]. Our results further suggested that SAFFE can discriminate functional status between a cohort of functionally intact older adults and persons meeting frailty criteria (albeit, we did not evaluate balance or falls in any of our participants). As mentioned above, in functionally intact (but not frail) individuals, SAFFE showed significant correlations to both LLFDI and PROMIS (except PROMIS-MH) scores. For all participants, correlations of submeasures within SAFFE (eg, SAFFE FF, SAFFE AL) were weaker (–.67 < *r* < –.33; .33 < *r*<.67). SAFFE scores also did not significantly correlate with either mobile phone gait speed or physical performance battery measures. Previous studies also have demonstrated weak correlation between SAFFE scores and accelerometer-based activity measures [55].

PROMIS-GH evaluates individual physical, mental, and social health domains, and is thus a more all-encompassing view of health status [56]. PROMIS-PF is a shorter, 10-question instrument that assesses individual physical health capacity without requiring a lengthy physical function instrument [23]. Compared to LLFDI and SAFFE, which were developed specifically for use in older populations, PROMIS-GH and PROMIS-PF assessments were developed for general adult populations [23,57]. Both of these instruments have previously been validated in a large, cross-sectional sample of independently dwelling US adults [23,58,59], as well as persons with chronic pelvic pain [60], cancer [61,62], or in preparation for surgical procedures [63]. Our results suggest that PROMIS-PF and PROMIS-PH can discriminate functional status capacity between a cohort of intact older adults and persons meeting frailty criteria. As mentioned above, in functionally intact (but not frail) individuals, we noted significant correlations between PROMIS and both LLDFI and SAFFE measures. PROMIS-GH and PROMIS-PF were also significantly correlated for functionally intact individuals, as were multiple physical performance battery measures. These findings suggested that PROMIS measures of physical capacity accurately reflected observed physical function in functionally intact individuals. However, in frail individuals, PROMIS measures had no significant correlations with all other metrics we quantified except for mobile phone-derived step count, LLFDI ALEF, and SAFFE AL.

A variety of physical performance measures have been adapted for clinic use, including the 4 Meter Walk [64], the timed Get Up and Go test [65], and the F8W test [29]. Both the timed Get Up and Go and F8W tests focus on older populations and have been used to assess community dwelling older adults and individuals with Parkinson’s disease. The 4 Meter Walk was developed for persons ranging from 7 to 85 years of age, and is a validated functional measure in persons with peripheral arterial disease [66] and cerebrovascular disease [67]. Our results demonstrated that all of these gait-associated performance batteries reliably distinguished between functionally intact older adults and older adults meeting frailty criteria. We also noted high correlations across these physical performance tests in both functionally intact and frail individuals. However, none of these measures correlated well with our mobile phone-derived activity and gait metrics.

### Mobile Phone-Derived Gait Metrics Reflect Different Aspects of Physical Function

In functionally intact individuals, there was little correlation between activity and gait metrics measured by mobile phone and participant responses to the LLFDI, SAFFE, or PROMIS instruments, or to physical battery performance. Similarly, Bland-Altman plots revealed that mobile phone-based metrics of physical activity and gait speed measured different aspects of physical capacity compared to LLFDI, SAFFE, PROMIS, or physical performance batteries. In functionally intact individuals, activity count, daily activity time budget, step count, and gait speed may undergo significant variation within a single individual as well as across many individuals. In other words, these particular functional metrics have considerable dynamic range. By contrast, survey- and physical performance-based instruments are well known to demonstrate ceiling effects in community dwelling individuals [68,69]. Thus, our functionally intact cohort may demonstrate few and weak correlations between mobile phone-based measures of physical activity and survey- or performance battery-based measures of the same, while simultaneously observing more and greater correlations when comparing measures known to have ceiling effects. We observed precisely this finding in our study.

However, functional measures characterizing frail individuals are far less likely to be influenced by ceiling effects. The decreased dynamic range and increased variability in functional status characteristic of frailty suggest that fewer correlations between different measures of physical capacity should occur in frail individuals. We noted this finding in our study as well. Finally, we noted significant correlations between step count (measured by mobile phone) and SAFFE activity restriction and PROMIS-PF. Mobile phone-derived gait metrics may estimate both activity restrictions and overall physical health (as well as gait speed, step count, and activity status) in older adults as they progress through stages of functional loss and ultimately become functionally impaired.

### Limitations

We recognize several limitations in this study, mostly regarding participant characteristics. Our desire to test cognitively intact individuals with functional impairments significantly limited our participant pool. While we ultimately envision that this technology will be used by cognitively impaired persons, for validation purposes we wanted to ensure that group differences could be attributed mostly to functional differences. We did not enroll a large group of cognitively intact individuals with functional deficits; however, given our effect size, we had adequate statistical power for discrimination. Our functionally intact group, self-selected from persons enrolled in a UNMC fitness program, sampled more health literate, financially secure, and higher educated individuals compared to community averages. We also did not quantify additional confounds, including medical comorbidities and pharmacotherapy. However, adjustment of study outcomes for these factors would likely have had only minimal impact on study outcome. Not surprisingly, we continued to note variable participant adherence for keeping the mobile phone during the study. While some participants successfully carried the phone and collected data for almost an entire 24-hour time frame, other individuals wore the phone for 10 hours or less. However, in practice, if individuals were to only collect data for brief, random periods each day, over longer time periods they would produce significant and robust datasets suitable for functional inference.

Given the worldwide ubiquity of mobile phone technology, and decreasing costs associated with mobile phone ownership, this study suggests that future healthcare systems should consider leveraging patient mobile phones to collect data associated with individual functional status (respecting patient privacy and autonomy), develop patient functional exemplars, and refine algorithms that not only calculate activity and gait functional metrics as above, but further identify within-individual acute and subacute functional changes in a reliable, robust, and efficient manner. This approach to population-wide healthcare is in its infancy, but already there is highly promising data suggesting that accurate knowledge of individual day-to-day patterns of behavior and functional status can be used to make rapid and accurate diagnoses of acute disease states [70]. Mobile phones also measure lifespace (an independent metric strongly associated with clinically important healthcare outcomes) [71,72] with high accuracy [73]. Ultimately, integrating these approaches into a comprehensive patient care platform that includes caregiver, decision making, and medication support may lead to significant improvements in patient quality of life, decreased healthcare spending, and improved care outcomes in persons with chronic illnesses, such as Alzheimer’s disease.

## Appendix

Multimedia Appendix 1 Bland-Altman plots demonstrate that mobile phone-based measures of activity and gait are independent of functional measures obtained from LLFDI, SAFFE, PROMIS PF, PH, and MH, and a physical performance battery. Matrix array depicting pairwise interactions between different study metrics. Matrix divided into regions (depicted by grey lower case letter in background) that group assay agreements for (a) all mobile-based metrics; (b) all LLFDI metrics; (c) all SAFFE metrics; (d) all PROMIS metrics; (e) all physical performance battery metrics; (f) mobile phone and LLFDI metrics; (g) mobile phone and SAFFE metrics; (h) mobile phone and PROMIS metrics; (i) mobile phone and physical performance battery metrics; (j) LLFDI and SAFFE metrics; (k) LLFDI and PROMIS metrics; (l) LLFDI and physical performance battery metrics; (m) SAFFE and PROMIS metrics, (n) SAFFE and physical performance battery metrics; and (o) PROMIS and physical performance battery metrics. Each plot is a mean-difference (Bland-Altman) plot comparing the metric listed at the top of the column to the metric listed at the left of the row. In each plot, RPC denotes reproducibility coefficient, CV is the coefficient of variation, and dotted lines depict 1.96 standard deviation from mean difference. Frail subjects denoted by red markers; functionally intact subjects denoted by blue markers. Note that with the exception of the LLFDI overall and LLFDI basic comparison, and the PROMIS PF and PROMIS PH comparison, none of the examined metrics appear to assess the same physical function constructs.

Multimedia Appendix 2 CONSORT checklist.

## Abbreviations

ALEF: advanced lower extremity function
ANOVA: analysis of variance
BMI: body mass index
F8W: Figure-of-8 Walk
LLFDI: Late Life Function and Disability Instrument
PROMIS: Patient Reported Outcomes Measurement Information System, short form version 1.0
PROMIS-GH: Patient Reported Outcomes Measurement Information System Global Health, short form version 1.1
PROMIS-MH: Patient Reported Outcomes Measurement Information System Mental Health
PROMIS-PF: Patient Reported Outcomes Measurement Information System Physical Function
PROMIS-PH: Patient Reported Outcomes Measurement Information System Physical Health
REDCap: Research Electronic Data Capture
SAFFE: Survey of Activities and Fear of Falling in the Elderly
UEF: upper extremity function
UNMC: University of Nebraska Medical Center
